# Lemierre’s Syndrome: A Rare but Resurging Disease

**DOI:** 10.7759/cureus.99895

**Published:** 2025-12-22

**Authors:** Ashwin Subramonian, Hajer Bdiri, Danial Bajwa, Clara Fong, Rabiu Momoh

**Affiliations:** 1 Internal Medicine, Medway Maritime Hospital, Kent, GBR; 2 Critical Care Medicine, Medway Maritime Hospital, Kent, GBR; 3 Critical Care, Medway Maritime Hospital, Kent, GBR

**Keywords:** case report, cavitatory pulmonary lesions, fusobacterium necrophorum, lemierre’s syndrome, microbiology, rare diseases, resurging disease, septic emboli, thrombosis of the internal jugular vein

## Abstract

Lemierre’s syndrome is a rare but life-threatening condition characterized by septic thrombophlebitis of the internal jugular vein (IJV) secondary to oropharyngeal infection, most commonly caused by *Fusobacterium necrophorum*. We report the case of a healthy 22-year-old male patient who presented with a sore throat, right-sided neck swelling, and sepsis. Blood cultures revealed *F. necrophorum*. Imaging confirmed IJV thrombosis and multiple cavitating pulmonary nodules consistent with septic emboli. The patient was managed with intravenous ceftriaxone and metronidazole, vasopressor support, and temporary anticoagulation. He made a recovery and was discharged home to complete an extended course of antibiotics. This case highlights the importance of early recognition of Lemierre’s syndrome in young adults presenting with oropharyngeal infection and systemic features of sepsis. Prompt diagnosis, prolonged anaerobic antibiotic therapy, and multidisciplinary input are vital to favorable outcomes.

## Introduction

Lemierre’s syndrome, sometimes called the “forgotten disease,” is a rare but serious complication of throat infections. It occurs when bacteria spread from the throat to a vein in the neck, causing a blood clot and infection that can travel to other parts of the body, often the lungs. The main culprit is usually a bacterium called *Fusobacterium necrophorum*, which normally lives in the throat. This condition mostly affects healthy young adults and can be life-threatening if not treated quickly [1].

Doctors are now seeing more unusual cases where the infection starts in places such as the ear, salivary glands, or stomach, and sometimes involves different bacteria such as *Staphylococcus aureus* or *Pseudomonas* [2].

First described in 1936, Lemierre’s syndrome almost disappeared after antibiotics became common, but it has returned in recent years, possibly because of changes in antibiotic use and resistance [3]. Studies show Lemierre’s syndrome is very rare, with only about three to six cases per million people each year. It is most common in teenagers and young adults, where rates can reach about 14 cases per million. In the past, it was even rarer, around one case per million, but reports suggest the numbers have roughly doubled over the last 20 to 30 years [4]. This case report adds to growing evidence about this rare but dangerous condition.

## Case presentation

A 22-year-old healthy male patient presented to a district general hospital’s emergency department with a six-day history of sore throat, right-sided neck swelling, and cough. His community general practitioner had managed him as a case of viral tonsillitis without improvement. He had no concerning travel history, but he reported a partner with similar symptoms.

On arrival, he was tachycardic (heart rate: 117 beats/minute), hypotensive (blood pressure: 99/53 mmHg), afebrile (36.6°C), and maintaining pulse oximetry saturation values at 100% on room air. He was alert and oriented. Chest and cardiac examinations were unremarkable, but there was a firm, tender swelling over the right submandibular and cervical region without tonsillar exudate or uvular deviation.

**Figure 1:**
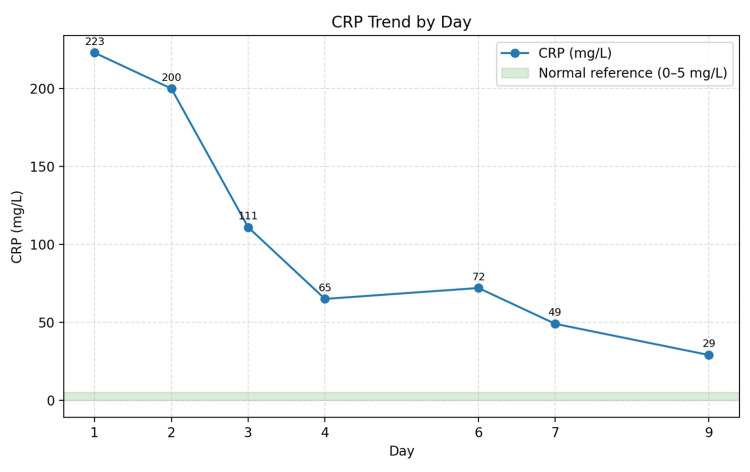
Serial serum C-reactive protein (CRP) findings.

**Figure 2:**
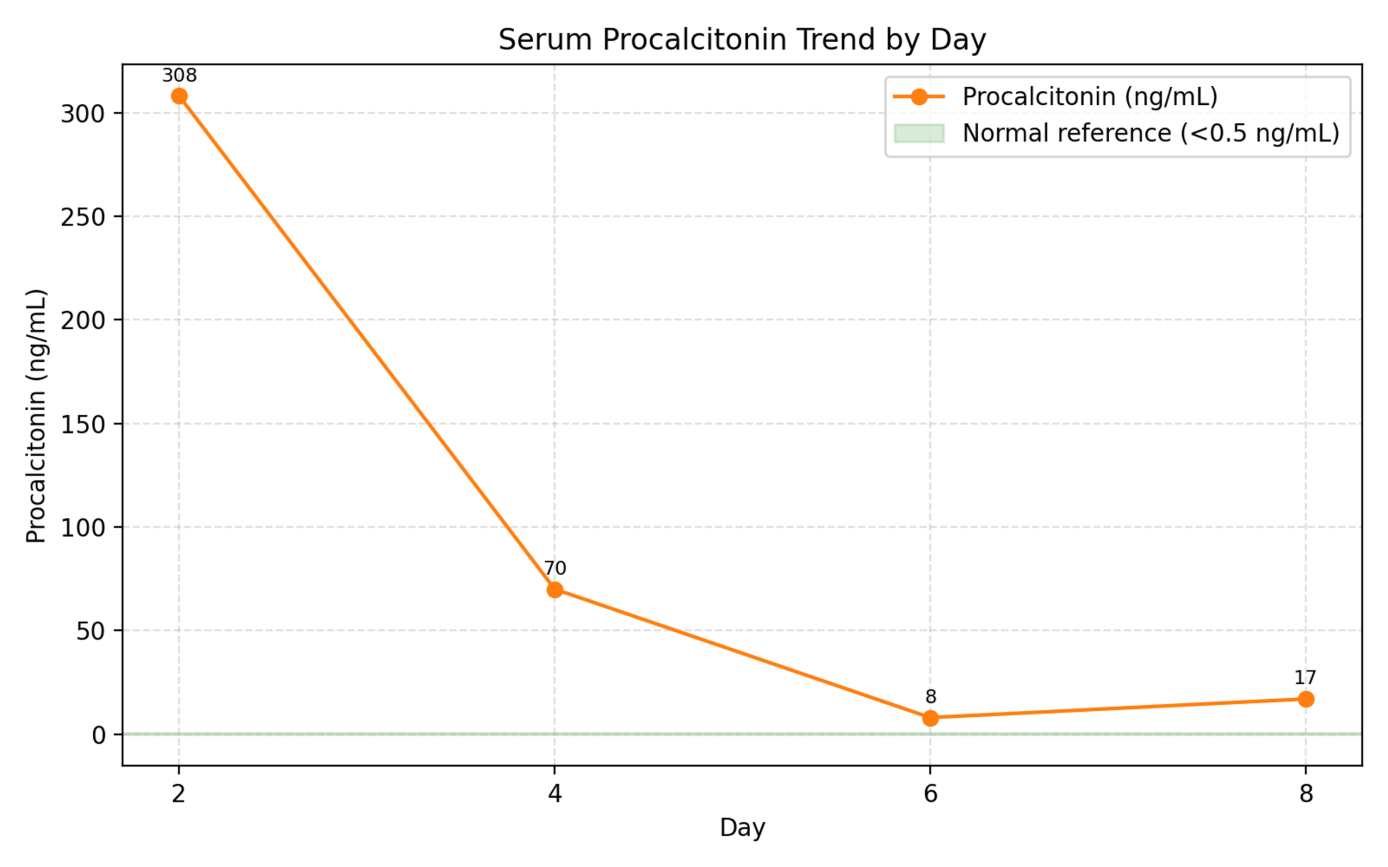
Serial procalcitonin findings.

**Figure 3:**
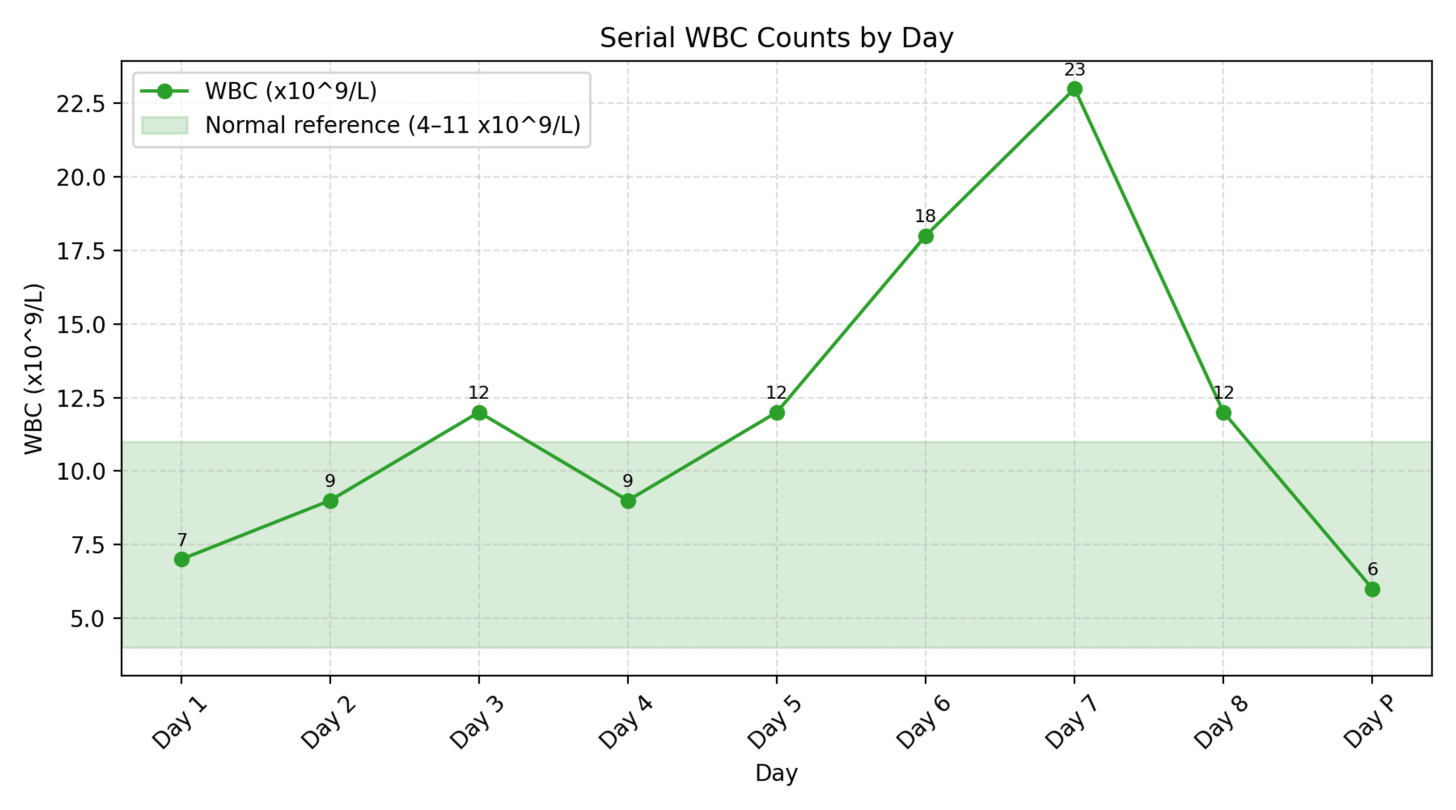
Serial white blood cell count (WBC) studies.

**Figure 4:**
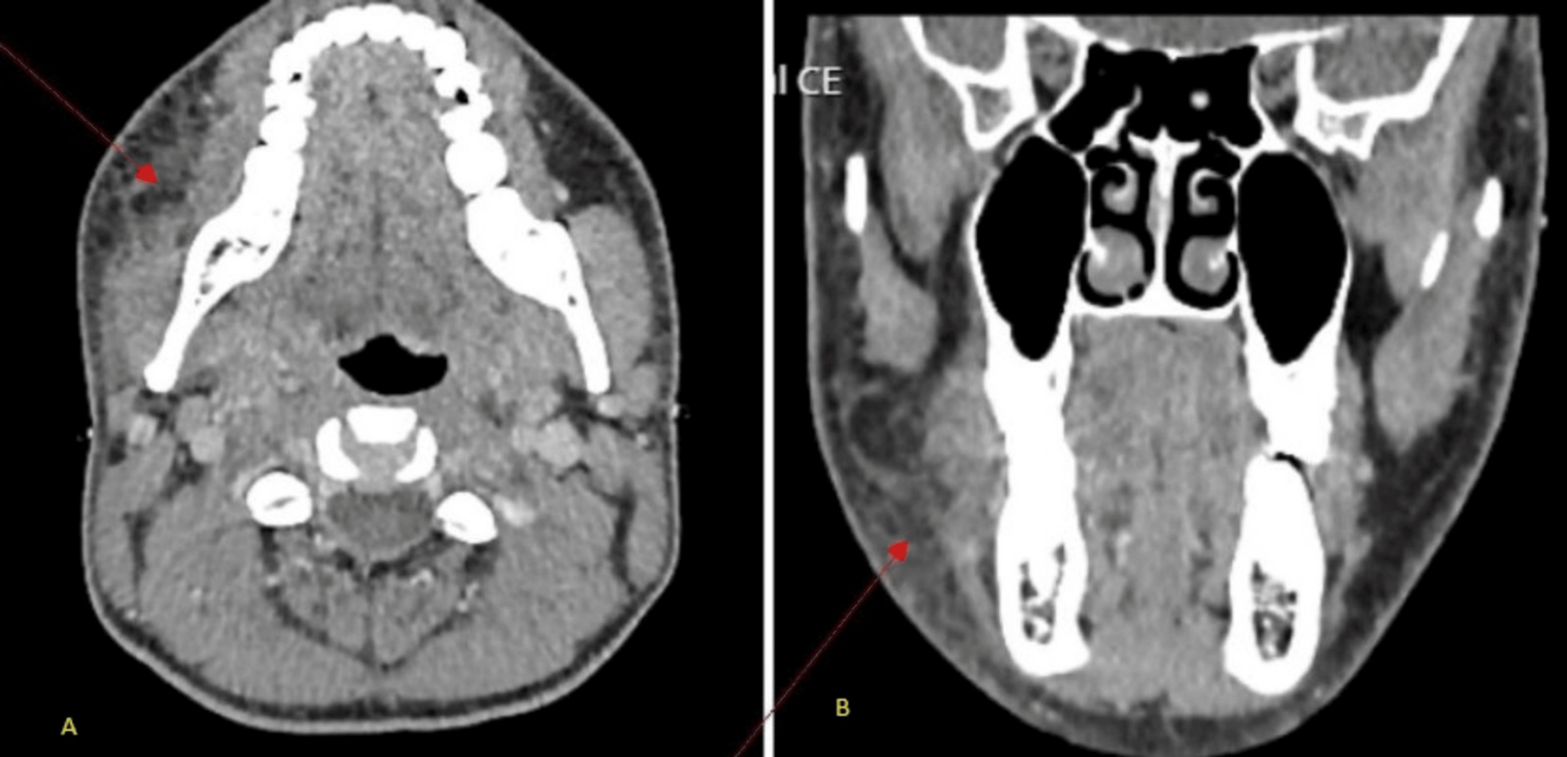
Infective soft-tissue swelling on the right side of the neck (marked with red arrows).

**Figure 5:**
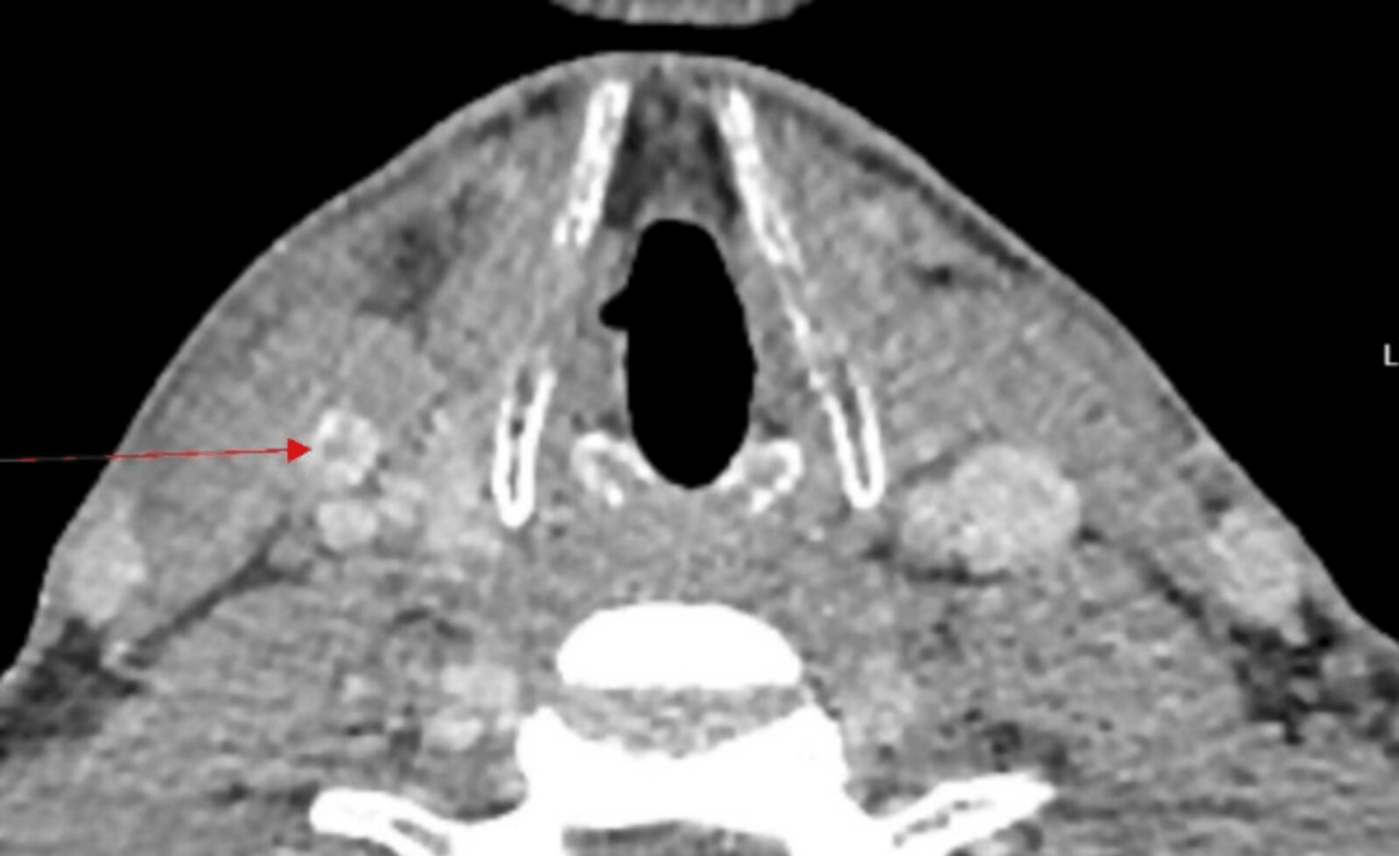
Transverse CT of the neck suggesting right internal jugular vein thrombophlebitis.

**Figure 6:**
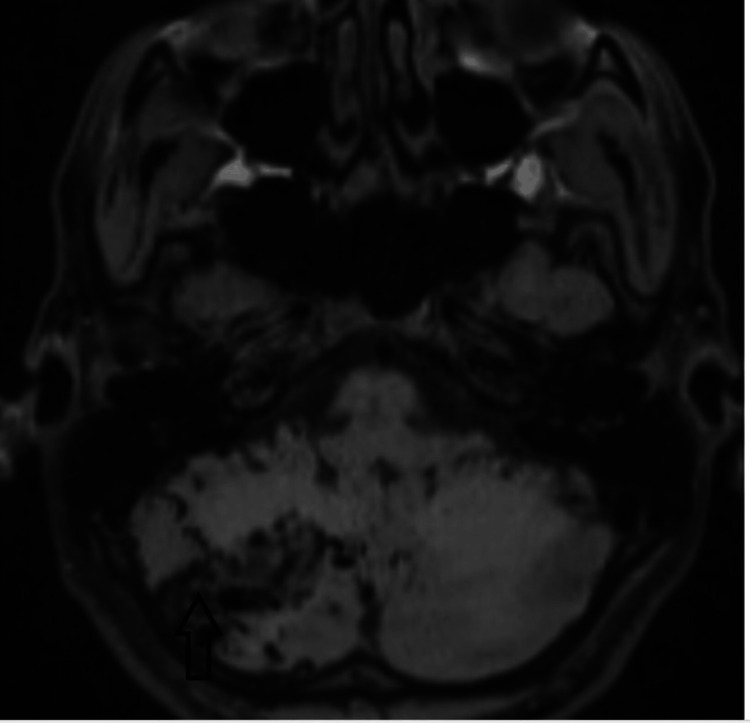
Incidental right cerebellar hemispheric hemangioma found on an MRI head scan study.

Laboratory Investigations done at admission are presented in Table 1. Serial infection marker study results are presented in Figures 1-3. A contrast-enhanced CT of the neck showed diffuse soft-tissue swelling compatible with cellulitis but no abscess (Figure 4). He was admitted to a high dependency unit (HDU) for vasopressor support and commenced on intravenous fluids, per oral clindamycin 300 mg QDS, and IV ceftriaxone 2 g BD. Following a microbiology unit review of the case and a positive finding of *F. necrophorum* from blood culture studies, his antibiotics were rationalized to IV ceftriaxone and IV metronidazole 500 mg TDS for a suggested total duration of six weeks. A repeat CT of the neck and thorax (day three) demonstrated right internal jugular vein (IJV) thrombosis (Figure 5), multiple cavitating pulmonary nodules (septic emboli), bilateral pleural effusions, and splenomegaly. He was anticoagulated with IV heparin following a hematology consultation. However, anticoagulation was discontinued after MRI/MRV demonstrated thrombus resolution and an incidental finding of right cerebellar hemispheric arteriovenous malformation (Figure 6).

**Table 1:** Laboratory investigations undertaken at the time of admission. CRP = C-reactive protein; AKI = acute kidney injury; eGFR = estimated glomerular filtration rate; AST = aspartate aminotransferase; INR = international normalized ratio; PCR = polymerase chain reaction; EBV = Epstein-Barr virus; RSV = respiratory syncytial virus; HIV = human immunodeficiency virus

Investigation	Result	Reference range	Comments
Serum CRP	223.4 mg/L	<5 mg/L	Markedly elevated
Platelet count	50 × 10⁹/L	150–400 × 10⁹/L	Thrombocytopenia
Creatinine	222 µmol/L	60–110 µmol/L	AKI stage 2
eGFR	32 mL/minute/1.73m²	>90 mL/minute/1.73m²	Reduced – consistent with AKI
Bilirubin	39 µmol/L	<21 µmol/L	Elevated
AST	69 U/L	<40 U/L	Suggests potential hepatic involvement from septic emboli/toxemia
INR	1.4	0.8–1.2	Suggests mild synthetic liver dysfunction
Blood lactate	3.5 mmol/L	0.5–2.2 mmol/L	Elevated
Respiratory viral PCR (EBV, COVID-19, influenza A/B, RSV)	Negative	—	
Blood culture	Grew *Fusobacterium necrophorum*	—	Confirms classic Lemierre’s syndrome pathogen
Serological tests (HIV, hepatitis)	Negative	—	Excludes HIV and viral hepatitis as contributing factors

Maxillofacial and dental reviews excluded odontogenic or salivary gland causes (normal orthopantogram studies). Echocardiography revealed biventricular functions without vegetations. The patient improved clinically and biochemically and was discharged after a nine-day hospital stay with outpatient parenteral antimicrobial therapy for three additional weeks.

## Discussion

This case demonstrates the classical triad of Lemierre’s syndrome: a primary oropharyngeal infection, septic thrombophlebitis of the IJV, and metastatic septic emboli, most commonly to the lungs. *F. necrophorum* is the predominant causative organism, accounting for nearly 80% of cases [5]. Since the advent of antibiotics, Lemierre’s syndrome became rare, but recent literature suggests a modest resurgence, primarily affecting healthy young adults [6].

The pathogenesis of Lemierre’s syndrome involves bacterial invasion of the lateral pharyngeal space. *F. necrophorum* produces leukotoxins and hemagglutinins that cause endothelial damage (thrombosis), platelet aggregation (thrombocytopenia), and septic emboli [5]. Although *F. necrophorum* is the most common organism, other anaerobes such as *Fusobacterium nucleatum* [7], *Bacteroides fragilis* [8], and *Streptococcus anginosus *[9] have also been implicated.

Pulmonary septic emboli occur in approximately 80-85% of patients, while hepatic, renal, and central nervous system metastases are less common but well-documented [10]. Kuppali et al. (2012) described a right frontal lobe brain abscess in a case report about Lemierre’s syndrome [5]. Our patient demonstrated multiple cavitating pulmonary nodules consistent with embolic dissemination, a hallmark of the syndrome. Diagnosis requires a high index of suspicion, especially in young, otherwise healthy individuals presenting with pharyngitis, neck swelling, and sepsis. Imaging (CT or MRI) confirming IJV thrombosis and growth of *F. necrophorum* in blood cultures are diagnostic hallmarks [6].

Gomide et al. (2022) touted that they had described the first case of Lemierre’s syndrome in a 52-year-old patient following a solid organ transplantation and on immunosuppressant therapy [11]. Risoud et al. (2015) described an atypical case of Lemierre’s syndrome associated with thrombophlebitis of the facial and anterior jugular veins [12]. Caruso et al. (2021) reported superior ophthalmic vein thrombophlebitis in a case of Lemierre’s syndrome caused by an odontogenic infection (*Streptococcus intermedius*) resistant to clindamycin [13]. Root et al. (2013) described a case of Lemierre’s syndrome in a 10-month-old child that was associated with a finding of a purulent pericardial collection causing cardiac tamponade and thrombotic stroke. They mentioned that their reported case was, probably, the youngest case description of Lemierre’s syndrome in the literature [14].

Other atypical literature descriptions of Lemierre’s syndrome are stated further here. Salam et al. (2024) described a fatal case of pyogenic liver abscess in a 51-year-old man with multiple septate hepatic masses and an associated partial splenic and portal vein thrombosis. He had a 16S polymerase chain reaction next-generation sequencing test done on an aspirate from the hepatic mass that returned positive for *F. nucleatum* [15]. Abdela et al. (2025) described a male in his 20s with recurrent acute otitis media who was assessed with bilateral IJV thrombosis and mastoiditis, confirmed via CT; he responded well to empiric antibiotics [2]. Burgdorf et al. (2024) described a penicillin-susceptible *S. aureus*-associated Lemierre’s syndrome in an adult male with chronic stomatitis and COVID-19 who presented with left facial and IJV thrombosis and bilateral pulmonary septic emboli [16]. Sapone et al. (2025) described a case of a 61-year-old with a parotid abscess (caused by *Pseudomonas aeruginosa*) that evolved into Lemierre’s syndrome with facial necrosis and was managed with IV antibiotics and anticoagulation [17]. Rangan et al. (2024) described a case of Lemierre’s syndrome caused by *Klebsiella pneumonia* in a poorly controlled diabetic [18]. Laurencet et al. (2019) described a case of atypical Lemierre’s syndrome caused by *F. nucleatum* in a patient who presented with lumbar pain and fever. A thrombosis of the iliac veins and abscesses in the right iliac and the left psoas muscles were diagnosed on CT, together with a right parapneumonic effusion and an L4-L5 spondylodiscitis [19].

The management of Lemierre’s syndrome involves prolonged antibiotic therapy with initial empirical broad-spectrum cover with clindamycin and ceftriaxone, and then de-escalated or narrowed to targeted therapy based on culture results. β-lactamase-resistant β-lactams (e.g., ceftriaxone) combined with metronidazole remain the cornerstone of treatment as per the literature [20]. A four-week antimicrobial course was administered to our patient following a multidisciplinary review that involved the maxillofacial, microbiology, and critical care units. Opinions on the role of anticoagulation vary in the literature, with no randomized data to guide practice. It may be reserved for extensive or propagating thrombosis or when there is cerebral sinus involvement [6]. The discontinuation of therapeutic anticoagulation in the index case was due to thrombus resolution on interval imaging and the finding of a cerebellar arteriovenous malformation. It was initiated initially due to the extensive IJV thrombophlebitis and septic pulmonary emboli.

With early recognition, appropriate antimicrobial therapy, and multidisciplinary care, the mortality rate from Lemierre’s syndrome has declined to below 5% as per the literature [20]. Our patient’s recovery following the prompt initiation of targeted antibiotics and supportive care underscores the importance of timely diagnosis and coordinated management.

## Conclusions

This case highlights that Lemierre’s syndrome continues to pose a serious and potentially fatal risk. Clinicians should consider it in young, otherwise healthy individuals presenting with persistent sore throat, neck pain or swelling, and systemic signs of sepsis, especially when thrombocytopenia and multi-organ dysfunction are evident. Early diagnostic steps, including CT imaging of the neck and chest and anaerobic blood cultures, are essential. Rapid initiation of broad-spectrum antibiotics covering anaerobes, followed by tailored prolonged therapy, is critical to achieving favorable outcomes.
